# Assessing the In Vivo Biocompatibility of Molecularly Imprinted Polymer Nanoparticles

**DOI:** 10.3390/polym14214582

**Published:** 2022-10-28

**Authors:** Samr Kassem, Stanislav S. Piletsky, Hasan Yesilkaya, Ozcan Gazioglu, Medhanie Habtom, Francesco Canfarotta, Elena Piletska, Alan C. Spivey, Eric O. Aboagye, Sergey A. Piletsky

**Affiliations:** 1 Nanomaterials Research and Synthesis Unit, Animal Health Research Institute, Agricultural Research Centre, Giza 12618, Egypt; 2 Department of Chemistry, Molecular Sciences Research Hub, White City Campus, Imperial College London, London W12 0BZ, UK; 3 Department of Respiratory Sciences, University of Leicester, Leicester LE1 7RH, UK; 4 School of Chemistry, University of Leicester, Leicester LE1 7RH, UK; 5 Department of Surgery and Cancer, Imperial College London, London W12 0NN, UK

**Keywords:** molecularly imprinted polymers, nanoparticles, cytotoxicity, biodistribution, blood–brain barrier

## Abstract

Molecularly imprinted polymer nanoparticles (nanoMIPs) are high affinity synthetic receptors which show promise as imaging and therapeutic agents. Comprehensive analysis of the in vivo behaviour of nanoMIPs must be performed before they can be considered for clinical applications. This work reports the solid-phase synthesis of nanoMIPs and an investigation of their biodistribution, clearance and cytotoxicity in a rat model following both intravenous and oral administration. These nanoMIPs were found in each harvested tissue type, including brain tissue, implying their ability to cross the blood–brain barrier. The nanoMIPs were cleared from the body via both faeces and urine. Furthermore, we describe an immunogenicity study in mice, demonstrating that nanoMIPs specific for a cell surface protein showed moderate adjuvant properties, whilst those imprinted for a scrambled peptide showed no such behaviour. Given their ability to access all tissue types and their relatively low cytotoxicity, these results pave the way for in vivo applications of nanoMIPs.

## 1. Introduction

Targeted drug delivery is the process in which drug molecules can be made to accumulate in specific tissues, typically facilitated by the use of antibodies or ligands with affinity for biomarkers which are either over-expressed or uniquely expressed within those tissues. Targeted delivery results in a higher concentration of the therapeutic agent at its site of action, facilitating lower doses and a corresponding reduction of side effects. In the past few decades, various types of nanoparticles (NPs), including micelles, nanogels and hydrogels, fibres and star polymers, have been developed to facilitate targeted delivery and improve the therapeutic efficacies of their payloads [1].

Polymeric particles of submicron size show particular promise as drug carriers. This is due to their ability to improve drug solubility, drug permeability through cell membranes, to facilitate controlled release, prolonged action, the possibility of oral delivery, and targeted delivery [2]. The polymer architecture of NPs dictates the carrier’s physiochemical properties, drug loading effectiveness, drug-release rate and biodistribution [3]. NPs smaller than 8 nm are cleared rapidly from the blood stream by the renal system and NPs larger than 200 nm are sequestered by the mononuclear phagocytic system in the liver and spleen [4,5]. NPs of 50–200 nm are considered to be good candidates for drug delivery, through a combination of high retention time, large capacity for therapeutic payloads, and benefiting from the enhanced permeability and retention (EPR) effect [6,7,8]. Given their highly controllable properties, the drug release profile of polymer NPs can be finely tuned either through gradual drug unbinding, the use of biodegradable polymers, or environmentally triggered release [9,10,11].

The demand for producing robust nanomaterials that mimic the recognition functions of antibodies has driven forward research in the field of molecular imprinting [12,13]. Molecular imprinting describes the creation of artificial molecular recognition sites in a synthetic polymer by the process of forming said polymer in the presence of a template molecule. Complementary interactions between polymer-forming components and the template species are preserved in their spatial arrangement by the polymerisation process and stabilised by cross-linking. Recent breakthroughs in nanomaterial engineering have enabled the manufacture of ‘plastic antibodies’: soluble molecularly imprinted polymer NPs (nanoMIPs) that have exquisite specificity and selectivity for their template molecules [14]. Newly developed methods for computer-controlled synthesis allow, for the first time, precise control over their production. This improves inter- and intra-batch consistency of particles, which can also be tailored for a range of specific functions [15]. Plastic antibodies represent an entirely new compound class which can now be deployed to address both extracellular protein targets (as an alternative to biological antibodies), and potentially also currently intractable intracellular proteins [16,17]. A highly desirable property offered by molecular imprinting is the fact that the binding target can easily be changed, simply by changing the template molecule. Cross-linked polymer NPs are highly stable, they can be highly hydrophilic, and in a biological environment they can swell and retain a high level of water/body fluids making them generally biocompatible [18].

Currently, however, our understanding of in vivo interactions of nanoMIPs remains under-developed, particularly with regard to how binding cavities affect internalisation, biodistribution and clearance. This has created a gap in knowledge and a substantial need for more research into the phenomena that occur once nanoMIPs have entered the blood stream and begun to interact with blood components, tissue and cell microenvironments. Herein, we report the synthesis of particles imprinted for trypsin, their labelling with a fluorescein derivative to track their mobility and study the clearance behaviour of these nanoMIPs in vivo. This target was selected as a protein which is not associated with the plasma membrane, and so these nanoMIPs should not accumulate on the surface of any particular cell type via specific binding mechanisms but rather show a distribution profile dependent on their size and surface properties. Following administration, these particles were present in a range of tissues samples including the liver, spleen, intestines and brain, and to exit the body via both faeces and urine. A brief study of the adjuvant properties of the nanoparticles was also performed, demonstrating that nanoMIPs specific for the transmembrane protein epidermal growth factor receptor (EGFR) showed elevated adjuvant properties compared to those imprinted for a scrambled control peptide.

## 2. Materials and Methods

### 2.1. Materials

All chemical reagents used in this project were obtained from Sigma-Aldrich Company Ltd./Merck Ltd. (Poole, UK) or Fisher Scientific Ltd. (Loughborough, UK) unless otherwise stated.

### 2.2. Solid Phase Preparation: Trypsin

**Figure 1:**
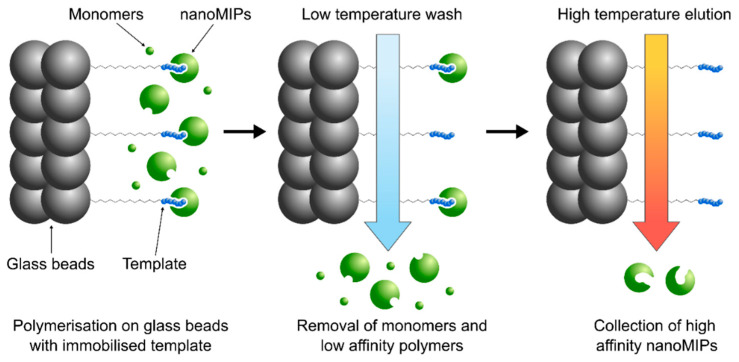
Schematics of the solid phase approach for the production of nanoMIPs.

NanoMIPs imprinted against trypsin were synthesised using a previously reported solid phase approach (Figure 1) [19]. The immobilisation of trypsin was performed as follows: glass beads (60 g) were boiled in NaOH (1 M, 200 mL) for 15 min, washed with water (5 × 200 mL), PBS (10 mM, pH 7.2, 2 × 100 mL), water (2 × 200 mL), acetone (1 × 100 mL) and allowed to dry. They were then incubated in 2% (*v*/*v*) (3-aminopropyl)triethoxysilane (APTES) in anhydrous toluene (50 mL) overnight at room temperature, protected from light. The beads were then washed with acetone (3 × 100 mL) and allowed to dry. The beads were then incubated for 2h in a 7% (*v*/*v*) solution of glutaraldehyde in PBS (50 mL, 10 mM, pH 7.4). The glass beads were then placed in a solution of trypsin (0.5 mg mL^−1^) in PBS (50 mL, 10 mM, pH 7.4) overnight. The beads were washed with water (3 × 100 mL) and dried under vacuum.

### 2.3. NanoMIP Synthesis

The following monomers were dissolved in water (100 mL): *N*-isopropyl acrylamide (NIPAm) (39 mg, 0.34 mmol), *N*-tert-butylacrylamide (33 mg, 0.26 mmol) in ethanol (1 mL), *N,N′*-methylenebis(acrylamide) (6 mg, 0.04 mmol), *N*-(3-aminopropyl)methacrylamide hydrochloride (6 mg, 0.03 mmol), acrylic acid (2.2 µL, 0.03 mmol) and fluorescein acrylamide (3 mg, 7 µmol) in ethanol (0.5 mL). The template-immobilised glass beads were added to the monomer solution, which was then bubbled with nitrogen for 20 min. Polymerisation was initiated through the addition of ammonium persulfate (48 mg, 0.21 mmol) and *N,N,N′,N′*-tetramethyl ethylenediamine (24 µL, 0.16 mmol) in water (800 µL). The mixture was shaken and incubated for 1 h before being transferred to a solid phase extraction cartridge fitted with a 20 µm polyethylene frit. Unreacted monomers and low affinity polymers were removed from the glass beads by washing with room temperature water (9 × 20 mL). High affinity polymers were collected with hot water (70 °C, 5 × 20 mL), reduced to 5 mL under vacuum and dialysed in water for 1 week using a 10 kDa cellulose membrane with regular change of water. These nanoMIPs prepared against trypsin were previously characterised via TEM imaging, showing an average diameter of 104 ± 19.5 nm (Figure S1) [19].

### 2.4. Animal Handling—Rats

Six-week-old pathogen-free Sprague Dawley rats (150 g bodyweight) were received from VACSERA laboratories (Egypt) and acclimatised for seven days after arrival prior to study at the Animal Health Research Institute (Giza, Egypt) laboratory animal house. Rodents were kept in an animal room at a regulated temperature (21–24 °C), humidity (30–45%), and light cycle (12 h light/dark). Food and water were available ad libitum. All in vivo studies with the NIH Guide for Care and Use of Laboratory Animals were approved by the Research Ethics Committee for Environmental and Clinical Studies (Protocol Number: 165429A).

### 2.5. Treatment of Rats with NanoMIPs

In vivo experiments were conducted on four groups (IV-100, IV-200, OR-100, OR-200) of 15 Sprague–Dawley male rats, each weighing 150 g. NanoMIPs were administered intravenously via the tail vein in groups IV-100 and IV-200 at two dilutions, 100 µg mL^−1^ and 200 µg mL^−1^ respectively. NanoMIPs were administered orally in groups OR-100 and OR-200 at two dilutions, 100 µg mL^−1^ and 200 µg mL^−1^ respectively. In both cases, the nanoMIPs were administered as a 300 µL solution in PBS, resulting in doses of either 200 or 400 µg kg^−1^ body weight, similar in dosage to previous in vivo studies on polyNIPAm nanoparticles [20,21]. These experiments were designed to determine biodistribution, clearance and cytotoxic properties of the nanoMIPs. The latter were tracked by fluorescence microscope in different harvested tissues (brain, liver, spleen) after isoflurane euthanisation for group IV-100 and IV-200, in addition to intestine for group OR-100 and OR-200, at time intervals 1, 24, 72 and 168 h post administration. Incidence of inflammation or body response was detected by measuring levels of Interleukin 1 beta (IL-1β) and C-reactive protein (CRP) in blood samples collected at time intervals 1, 24, 72 and 168 h post injection for groups IV-100 and IV-200, and at time intervals 2, 24, 72 and 168 h for groups OR-100 and OR-200. Quantitative analysis of NPs in urine and faeces was performed at time intervals 12, 24, 72 and 168 h post administration.

### 2.6. Fluorescence Imaging of Tissue

Liver, spleen and brain specimens were collected from all experimental groups, and additionally intestine specimens from groups OR-100 and OR-200. These samples were collected at time interval 1, 24, 72 and 168 h after injection of nanoMIPs for group IV-100 and IV-200 (IV, intravenous) and at time interval 2, 24, 72 and 168 h for groups OR-100 and OR-200 (OR, oral). These were then sectioned in a cryostat at 4 µm thickness, fixed in acetone for 5 min, washed in PBS three times and mounted into gelatin coated slides for examination under a fluorescent microscope (Olympus BX51) as previously described [22].

### 2.7. Histopathological Studies

Liver, spleen and brain specimens were collected from all experimental groups, and additionally intestine specimens from groups OR-100 and OR-200. These samples were collected at time intervals 1, 24, 72 and 168 h after injection of nanoMIPs for group IV-100 and IV-200, and at time interval 2, 24, 72 and 168 h for groups OR-100 and OR-200. Tissue sections were immediately fixed in 10% (*v*/*v*) formalin after necropsy and prepared for histopathological examination using the embedding method of standard paraffin. Sections of 3 μm thickness were cut and stained for light microscopic examination using hematoxylin and eosin (H & E), as previously described [23].

### 2.8. Detection of C-Reactive Protein (CRP), Interleukin 1-β (Il-1β)

Blood was drawn from the abdominal artery in animals anesthetised with inhaled isoflurane in a plain tube at time interval 1, 24, 72 and 168 h post injection of NPs for groups IV-100 and IV-200, and at time interval 2, 24, 72 and 168 h for groups OR-100 and OR-200. This blood was analysed for the detection of interleukin 1 beta (IL-1β) and C-reactive protein (CRP). Blood samples were allowed to coagulate at room temperature for 2 h before centrifuging for 20 min at 1000× *g*. Quantitative determination of IL-1β concentrations in serum was performed using an Quantikine Rat IL-1β immunoassay kit supplied by R & D systems, Inc. (Minneapolis, MN, USA) as previously described [24]. Quantitative determination of CRP concentrations in serum using BD^TM^, Rat CRP ELISA kit supplied by BD Biosciences, Inc. (San Diego, CA, USA) as previously described [25].

### 2.9. Quantitative Analysis of NPs in Urine and Faeces

Urine and faeces were collected for 12 h using a metabolic cage (Nalgene, Rochester, NY, USA) at time interval 12, 24, 72 and 168 h post administration. Urine and faeces samples were diluted with PBS, and fluorescence measured in triplicate using an excitation of 500 nm, and emission of 550 nm using a fluorescence microplate reader (Packard Instruments, Meriden, CT, USA). Serial dilutions of the nanoparticles with urine and faeces control samples were made to obtain a standard fluorescence intensity curve across the nanoparticles mass. The concentration of nanoparticles in the samples was measured using a calibration curve to convert fluorescence to mass of nanoparticles as previously described [26].

### 2.10. Detection of MIPs in Urine and Brain Tissue by TEM

Samples were collected for TEM analysis from group OR-200 at 24 h post administration. The collected urine samples were, respectively, centrifuged. The precipitates were then incubated for 30 min with 1 M HCl solution and centrifuged again. Pre-treated precipitates were washed with water three times, followed by water suspension, and dropped onto a detection grid of TEM JEOL (JEM-1400 TEM). Brain tissue samples were sliced to 1 mm thickness of slices and was prepared for TEM by glutaraldehyde and osmium tetroxide fixation, alcohol dehydrated and deposited in an epoxy resin. Microtome sections prepared with a Leica Ultracut UCT ultramicrotome at a thickness of around 500–1000 µm. Thin sections were stained with tolodin blue (1×), then sections were analysed using a Lica ICC50 HD camera. Ultra-thin sections were processed at a thickness of 75–90 µm and stained with uranyl acetate and lead citrate, then examined at candidate magnification by transmission electron microscope JEOL (JEM-1400 TEM). Images were captured at side mount configuration using a CCD optronic camera (Advanced Microscopy Techniques Corp., Danvers, MA, USA) with 1632 × 1632 pixel size [27].

### 2.11. Solid Phase Preparation: EGFR Peptides

Two EGFR peptides were selected: an EGFR-specific peptide previously identified during epitope mapping of EGFR (CGKLFGTSGQK), and a scrambled version of the same peptide (CGTKGKLQSGF) [28]. Both peptides possessed terminal cysteine for immobilisation purposes and glycine as a spacer. Solid phase preparation was performed as described by Piletsky et al. [29]. Glass beads (60 g) were boiled in NaOH (1 M, 200 mL) for 15 min, washed with water (5 × 200 mL), PBS (10 mM, pH 7.2, 2 × 100 mL), water (2 × 200 mL), acetone (1 × 100 mL) and allowed to dry. They were then incubated in 2% (*v*/*v*) (3-iodopropyl)trimethoxysilane (IPTMS) in anhydrous toluene (50 mL) overnight at room temperature, protected from light. The beads were then washed with acetone (3 × 100 mL) and allowed to dry. The beads were incubated overnight in a solution of peptide (0.1 mg mL^−1^) in borate buffer (50 mL, pH 8.5, 25 mM). The beads were washed with water (3 × 100 mL) and dried under vacuum. NanoMIP formation was then performed as in above ‘nanoMIP synthesis’ section.

### 2.12. Animal Handling—Mice

Mouse studies were done under the UK Home Office approved project (PP0757060) and personal licenses (I4FF857C1) and approved by the local ethic committee. The mice were kept in individually ventilated cages in a controlled environment. Food and water were available ad libitum.

### 2.13. Immunisation of Mice

To raise polyclonal antibody, 10–12 weeks old outbred CD1 mice were administered intraperitoneally with one of the following in a solution of PBS (100 µL, 10 mM, pH 7.0): ovalbumin (10 µg), ovalbumin (10 µg) with Imject Alum (33 µL, containing 40 mg mL^−1^ aluminium hydroxide and 40 mg mL^−1^ magnesium hydroxide, Thermo Fisher (Oxford, UK), ovalbumin (10 µg) with EGFR-specific nanoMIPs (40 µg), or ovalbumin (10 µg) with scrambled nanoMIPs (40 µg). A control group was treated with PBS alone. Each of these treatments were performed three times with two weeks intervals. For each treatment, between 8–10 mice were used. Two weeks after the last administration, blood was obtained with cardiac puncture under deep anaesthesia and the serum was collected by centrifugation. Until use, the serum was kept at −80 °C.

### 2.14. Quantification of Ovalbumin-Specific IgG Levels in Mouse Serum

Ovalbumin-IgG levels were determined by indirect ELISA using a commercial kit (Assay Genie, Dublin, Ireland) as per the standard protocol of kit. A 96-well microplate plate was pre-coated with antigen, and 100 µL of standard solutions added to wells in triplicate. Serum samples were diluted in PBS by a factor of 24,000. 100 µL of serum was added to the wells in triplicate, and the plate incubated at 37 °C for 90 min. The wells were then aspirated and washed with the included wash buffer three times. 100 µL of HRP-labelled antibody working solution was added to each well, and the plate incubated at 37 °C for 30 min. The wells were then aspirated and washed with the wash buffer five times. 90 µL of TMB substrate was added to each well, and the plate incubated at 37 °C for 15 min. 50 µL of stop solution was then added to each well, and the colour development, which is proportional to the target amount of sample captured in plate, was quantified at 450 nm in a microplate reader (LT-4500, Labtech, Heathfield, UK).

## 3. Results and Discussion

### 3.1. Biodistribution of NanoMIPs

To study the distribution of nanoMIPs in vivo, two batches of nanoMIPs were administered to 60 Sprague–Dawley male rats. These rats were separated into four equal groups (IV-100, IV-200, OR-100, OR-200), and administered nanoMIP solutions as described above. In all cases, the administered nanoMIPs were imprinted for trypsin. The intention of this study was to investigate the biodistribution of nanoMIPs in the absence of specific binding mechanisms, rather than targeting one particular biomarker or tissue type. For this reason, trypsin was selected as a template because it is a non-cell-surface protein that has been extensively characterised as a template for imprinting [19,30,31]. Furthermore, the nanoMIPs were not loaded with any therapeutic payload such that the cytotoxicity of the nanoMIPs themselves could be assessed without interference from pharmaceutically active substances. Due to the nature of the solid phase nanoMIPs synthesis route, a template molecule is required in order to generate polymers, so non-imprinted polymers were not a viable alternative.

The rats treated via the oral route were tested to verify whether adsorptive endocytosis can be exploited via oral delivery of nanoMIPs to the blood stream. NanoMIPs were tracked by fluorescence microscopy in different harvested tissues (brain, liver, spleen) to validate their internalisation. In general, the liver and spleen are major sites for accumulation of NPs regardless of the exposure route. The presence of NPs in brain tissue, however, would not necessarily be expected due to the presence of the blood–brain barrier (BBB).

**Figure 2:**
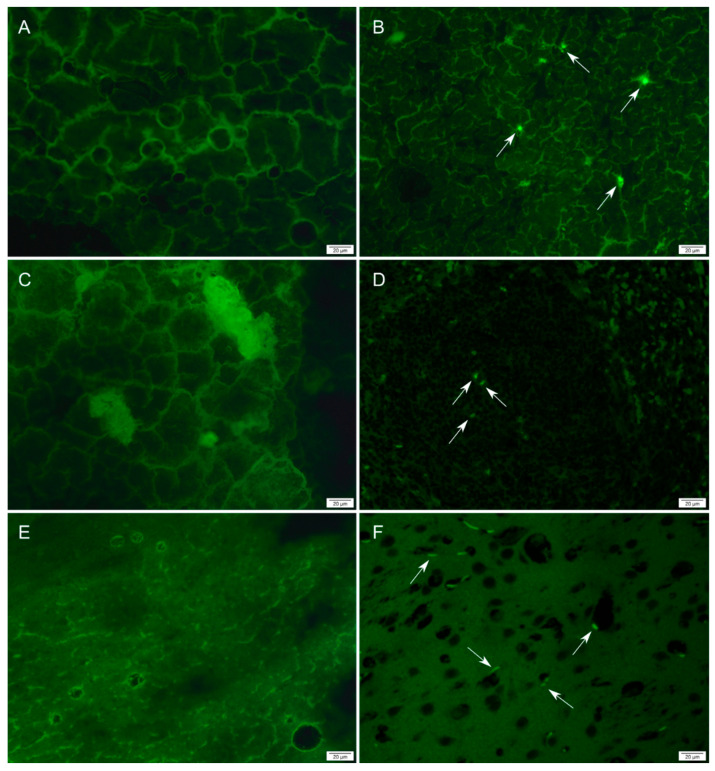
(**A**) Liver control; (**B**) Liver, IV-100 at 48 h; (**C**) Spleen control; (**D**) Spleen, IV-100 at 1 h; (**E**) Brain control; (**F**) Brain, IV-100 at 1 h. Arrows indicate examples of nanoMIPs and aggregates. Scale bar = 20 µm.

Fluorescent nanoMIPs were detected in all harvested tissue samples, including brain, liver and spleen tissue (Figure 2), as well as in the intestines of rats treated with OR-100 and OR-200 (Figure S2). Particles and their aggregates were seen as discrete objects in the tissue. The fact that nanoMIPs can be detected in harvested organs demonstrates their suitability for being developed into a convenient approach to drug delivery that does not require intravenous injections. The adsorption-mediated transcytosis of NPs through a cell is also useful for drug transport into the brain [32]. The BBB is impenetrable for the majority of common drug molecules, as it is poorly permeable to both small and large molecules except for a limited range of lipophilic molecules with 400–500 Da in mass [33]. Current strategies for developing polymeric drug carrier are based on using water-soluble biocompatible and biodegradable copolymers such as poly (butyl cyanoacrylate), poly (lactic acid) and poly (lactide-co-glycolide) [34]. Often polymer NPs are conjugated with transferrin, lactoferrin, antibodies to transferrin, APOE and TAT peptides to increase permeability across the BBB [35]. The presence of nanoMIPs in brain tissue is promising, as it implies that MIPs may be able to transport drugs across the BBB for the treatment of diseases of the central nervous system.

**Figure 3:**
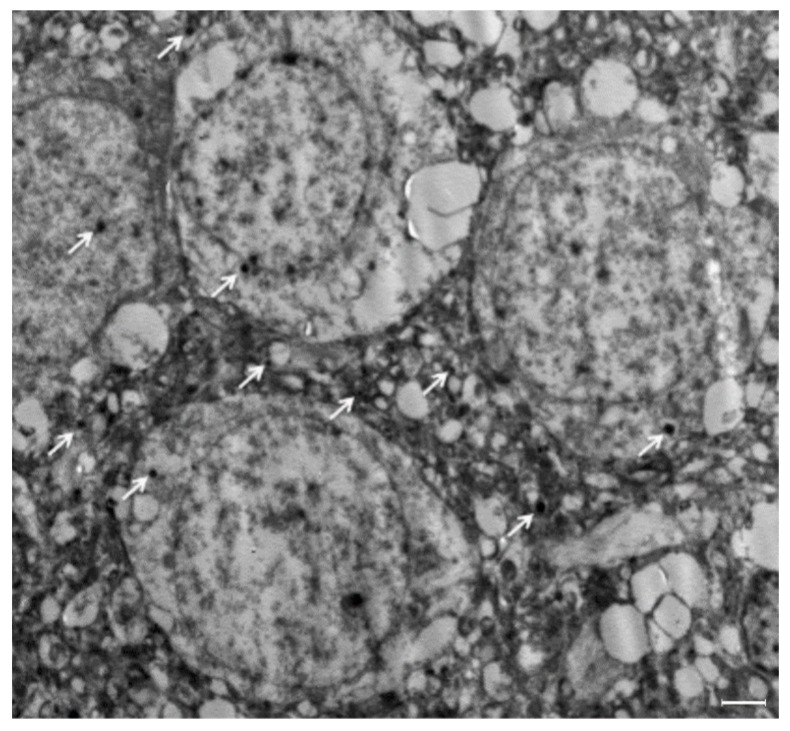
TEM images show nanoMIPs in the brain parenchyma. Brain sample was collected from group OR-200, 2 h post administration. NanoMIPs (identified by arrows) are visible as dark, electron dense areas. Scale bar = 2 μm.

To verify the presence of nanoMIPs within brain tissue, we have performed additional experiments using TEM imaging. As in the case of fluorescent analysis, discrete nanoMIPs with sizes of 100–200 nm were detected in brain tissue (Figure 3). The fact that nanoMIPs can cross BBB without osmotic treatment or relying on active transport is highly important for developing new tools for targeted drug delivery.

### 3.2. In Vivo Clearance of NanoMIPs

There are two opposing considerations regarding NP clearance that influence the design of NPs used in drug delivery. Overall, prolonged circulation of NPs in blood is useful for controlled release of drugs over a significant period of time. However, nanoparticles with greater retention times are affiliated with higher likelihood of toxicity and immunological response [36]. Clearance of NPs typically depends on dose, size and surface chemistry [37].

It is expected that particles exhibiting a size range of <10 nm would primarily be excreted via a renal pathway, while hepatic and splenic clearance is typically important for particles < 500 nm through either macrophage clearance or splenic fenestration [38]. For the majority of NP systems, the most prominent mechanism of liver accumulation is phagocytosis by Kupffer cells [37]. It is believed that cross-linked polymers have poor biocompatibility, and for this reason, they are believed to be inferior to biodegradable nanocomposites [39]. In addition, biodegradable polymers have their own issues related to poorly controlled distribution and potential toxicity of polymeric fragments [40] 

**Figure 4:**
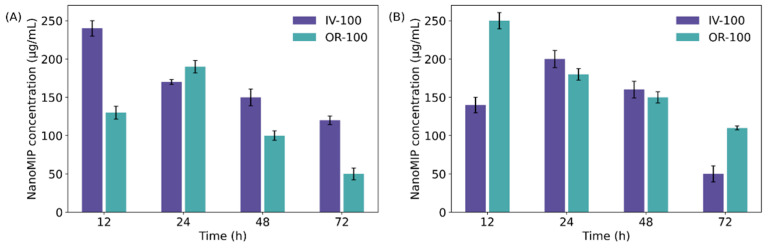
Concentration of nanoMIPs in rat excretion for groups IV-100 and OR-100. (**A**) Concentration of nanoMIPs in urine; (**B**) Concentration of nanoMIPs in faeces.

**Figure 5:**
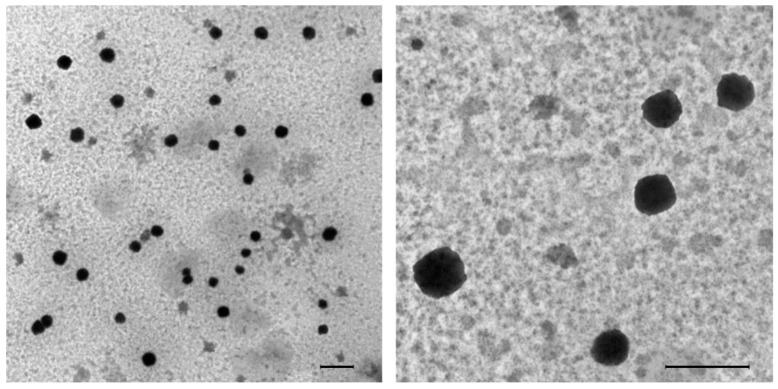
TEM images of nanoMIPs in urine of group OR-200. Scale bar = 500 nm.

To study the clearance of nanoMIPs, urine and faeces were collected at specific time intervals (12, 24, 72 and 168 h post-administration for all groups), suspended in PBS and their fluorescence measured as described above. For group IV-100 the concentration of nanoMIPs in urine showed an initial spike at 12 h, and gradually declined over the following 72 h (Figure 4A) and were no longer observed after 168 h. For group OR-100, the nanoMIPs concentration in urine reached a maximum at 24 h and declined over 72 h, also completely disappearing by 168 h. In faeces the situation was reversed, with group OR-100 showing a maximum concentration at 24 h, and the concentration in faeces of IV-100 showing gradual decline over 72 h (Figure 4B). As in the case of urine, no particles were detected in faeces after 168 h. In the higher concentration group IV-200, the majority of nanoMIP clearance was initially via the urine (12 h and 24 h) and then via the faeces (48 h and 72 h). OR-200 showed a more similar profile to OR-100 with the majority initially excreted via the faeces within 24 h (Figure S3). TEM imaging of urine from group OR-200 also demonstrates the presence of nanoMIPs (Figure 5). Of course, the absence of nanoMIPs from urine samples post 168 h does not preclude the presence of remaining nanoMIPs within the rats. For this, fluorescence analysis of tissue samples must be performed at later time points and is not expanded upon within this work.

This behaviour is in line with typical clearance profile observed for other type of NPs [41]. Polymeric nanoparticles of 155–240 nm diameter were previously shown to undergo renal clearance, attributed in part to their highly negative charge. The authors proposed that the particles were taken up by the epithelial cells of the proximal convoluted tubules (PCT) via endo- or micropinocytosis [42]. The nanoMIPs prepared in this study possess acrylic acid as a negatively charged monomer and were of similar size, implying the same mechanisms may be involved here. It is also possible that renal clearance of nanoMIPs is the result of adsorptive transcytosis, as previously discussed. Finally, renal clearance may be possible due to the soft structure of the nanoMIPs; it has been previously shown that if a force close to the renal filtration pressure is applied to soft NPs, they can pass through pores more than 10 times smaller than their size [43]. Which effect is more prominent would require further investigation by synthesising NPs with different charges, sizes, degree of cross-linking and stiffness, and then studying their release from the body.

### 3.3. Cytotoxicity of NanoMIPs

The criteria for polymeric NPs to act as imaging or delivery agents in vivo are that they must be stable, biologically inert, and non-toxic. Polymeric NPs can activate platelets, induce coagulation cascade events owing to activation of specific enzymes and activate the complement cascade, initialising rapid clearance of NPs by the mononuclear phagocytic system [44]. Evidence suggests that polymeric NPs likely have an indirect effect on adaptive immune responses, most likely through production of inflammatory cyto- and chemokines and promotion of dendritic cell maturation [45]. The signs of toxicity and severity caused by administration of NPs are usually dose dependent and differed according to route of administration [46]. In ex vitro experiments nanoMIPs have shown a cytotoxicity between 10 and 15% at concentrations up to 200 µM [47]. Very little is known however about toxicity of nanoMIPs in vivo, especially in relation to different organs. In particular, the exact contribution of endocytosis of NPs to cellular toxicity is poorly understood [40].

NPs are usually taken up by macrophages in macrophage-rich organs, such as the liver and spleen, which induces the release of cytokines from macrophages. Previous studies have shown that systemic exposure to NPs may increase the levels of interleukin-6 (IL-6), monocyte chemoattractant protein-1 (MCP-1), and granulocyte-macrophage colony-stimulating factor (GM-CSF), as well as the number of T cells, mast cells, natural killer cells, endothelial cells and fibroblasts in animals treated with NPs [37].

**Figure 6:**
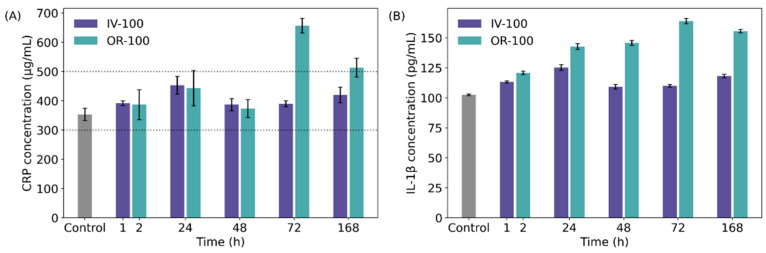
(**A**) CRP concentration in blood of groups IV-100 and OR-100. Dotted lines depict range of basal CRP concentrations in healthy rats; (**B**) IL-1β concentration in blood of groups IV-100 and OR-100.

To analyse nanoMIP cytotoxicity, serum samples were collected at time interval 1, 2, 24, 72 and 168 h after injection as described above. Two inflammatory biomarkers, C-reactive protein (CRP) and interleukin-1β (Il-1β) were detected in blood by ELISA kits to assess the inflammatory response caused by treatment with nanoMIPs. The level of CRP was observed to be only mildly elevated in response to nanoMIP treatment, in both groups treated intravenously and groups treated via oral administration (Figure 6A). The CRP concentrations within the rats treated intravenously with lower nanoMIPs concentrations (group IV-100) did not deviate significantly from the basal CRP concentration of healthy rats, which is typically between 300 and 500 µg mL^−1^ (Figure 6A) [48]. Only two time points of the orally treated group (OR-100) exceeded a concentration of 500 µg mL^−1^: most prominently OR-100 at 72 h (657 µg mL^−1^), falling again at 168 h (to 513 µg mL^−1^). Interestingly, samples from rats administered via oral administration (OR-100) but not intravenous administration (IV-100) have shown an increase of up to 60% in the level of Il-1β (Figure 6B). CRP and IL-1β levels were more elevated in groups treated with higher nanoMIPs concentrations (IV-200 and OR-200), with a more pronounced increase in CRP concentration in group IV-200 than OR-200 (Figure S4). We can conclude that during treatment with nanoMIPs, the levels of inflammatory biomarkers are dependent on both the concentration of the administered particles and on the route of administration, with oral administration showing a more pronounced effect on inflammatory biomarker concentration [49].

**Figure 7:**
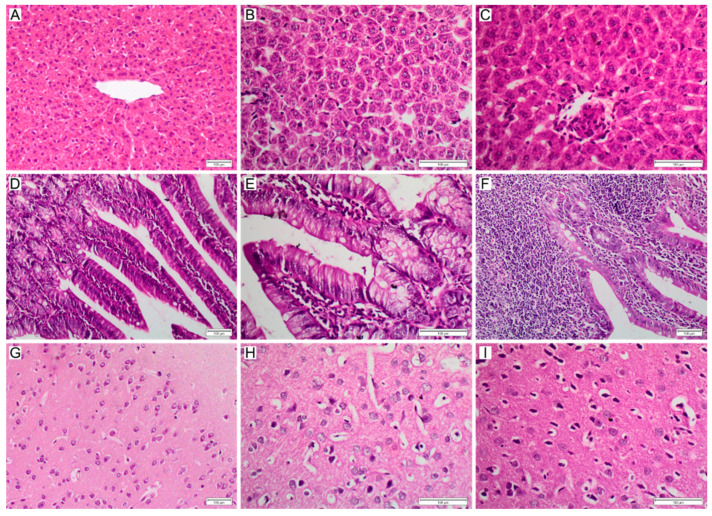
Histopathological changes induced by nanoMIPs over time. (**A**) Liver control; (**B**) Liver, IV-100 at 24 h; (**C**) Liver, IV-100 at 1 week; (**D**) Intestine control; (**E**) Intestine, OR-100 at 24 h; (**F**) Intestine, OR-100 at 1 week; (**G**) Brain control; (**H**) Brain, IV-100 at 24 h; (**I**) Brain, IV-100 at 1 week. Scale bars = 100 µm.

**Figure 8:**
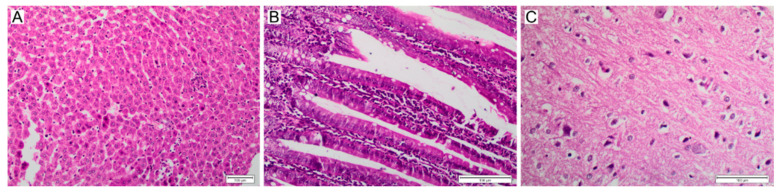
Histopathological changes induced by higher concentrations of nanoMIPs. (**A**) Liver, IV-200 at 1 h; (**B**) Intestine, OR-200 at 1 week; (**C**) Brain, OR-200 at 24 h. Scale bars = 100 µm.

Histopathological analysis was performed for several samples of harvested tissues (Figure 7), with the intention of identifying signs of inflammation, necrosis and other metrics of toxicity. The liver is considered to be the main organ affected as a systemic response to xenobiotic exposure in animals, primarily observed as a proliferation of mononuclear phagocytes. Liver sections for group IV-100 revealed only mild changes including mild lobular inflammation by lymphocytes, as well as Kupffer cells 1 h post injection (Figure 7B) and sinusoidal dilatation and necrotic focus with mild lobular inflammatory cell infiltration by lymphocytes and Kupffer cells at 1 week post injection (Figure 7C). Group IV-200 after 1 h revealed marked changes in liver histology with necrotic foci and mild lobular chronic inflammatory cell infiltration by lymphocytes and Kupffer cells (Figure 8A). Sections of the intestines for group OR-100 showed appearances within the limits of normal (Figure 7E). More significant damage is observed for intestine samples at the one week time point, including severe expansion of the lamina propria by chronic inflammatory cells including lymphocytes, plasma cells, and occasional eosinophils. There was also a mild increase in apoptosis within the surface epithelium (Figure 7F). Specimens exposed to the higher concentration of NPs administered orally (OR-200) showed architectural distortion including elongation of villi and proliferation of crypts, as well as increase in goblet cells (immune cells in the intestinal barrier) (Figure 8B). They also showed a mild to moderate expansion of the lamina propria by chronic inflammatory cells, mildly increased intraepithelial lymphocytosis and mildly increased apoptosis of the surface epithelium. Sections of brain tissue samples for group IV-100 showed only mild to moderate changes in response to NPs administration expressed in the form of astrocytes activation as part of the normal immune response, without neuronal damage (Figure 7H). For group OR-200, more noticeable neuronal damage was observed as neurofibrillary tangles and areas of neutrophil vacuolation (Figure 8C). 

Overall, it is possible to conclude that nanoMIPs administered at the lower concentration (100 µg mL^−1^) have only a small histologically observable effect on cells and tissues such as infiltration of inflammatory cells including mononuclear phagocytes, without evidence of severe inflammation, architectural changes, or necrosis. More pronounced signs of toxicity were observed in the higher dose groups (200 µg mL^−1^), with observable neuronal damage in the brain, coagulative necrosis in the liver, architectural distortion and severe inflammation in the bowel. This study has been done using short-term exposure regimens, and there is a need for further studies assessing the long-term effects of nanoMIPs, such as after chronic exposure.

### 3.4. Immunogenicity of NanoMIPs

As discussed above, there is growing interest in using nanoMIPs as drug delivery vehicles, and in most cases it would be undesirable for MIPs to promote immune responses against their payloads. This is particularly true in the case of protein and peptide-based therapies, as these payloads are more likely to act as antigens. However, adjuvant properties can also be exploited for the creation of new vaccines, and for adjuvant immunotherapy. Polymeric nanoparticles have already been proposed as carriers for immunomodulators in order to stimulate the immune response for enhanced cancer immunotherapy [50]. Targeted binding of polymer nanoparticles for the delivery of immunomodulators could potentially achieve even greater treatment efficacy.

To assess the adjuvant properties of nanoMIPs, mice were treated with nanoMIPs prepared against two peptides as described above. Of these two peptides, one is an epitope of epidermal growth factor receptor (EGFR), a transmembrane protein commonly overexpressed by cancer cells but also present on non-cancerous cells, being involved in the development and maintenance of healthy tissue [51] The second peptide is a scrambled version of the first, and hence is used as a non-specific control. NanoMIPs prepared against the EGFR epitope are hence more likely to interact with cell surfaces, resulting in longer retention time and potentially more pronounced adjuvant properties. During this experiment, mice were injected with ovalbumin alone, mixed with either of the two peptide-imprinted nanoMIPs, or with Alum Imject Adjuvant, a formulation of aluminum hydroxide and magnesium hydroxide that stimulates the immune response for antibody production procedures when mixed with other immunogens.

**Figure 9:**
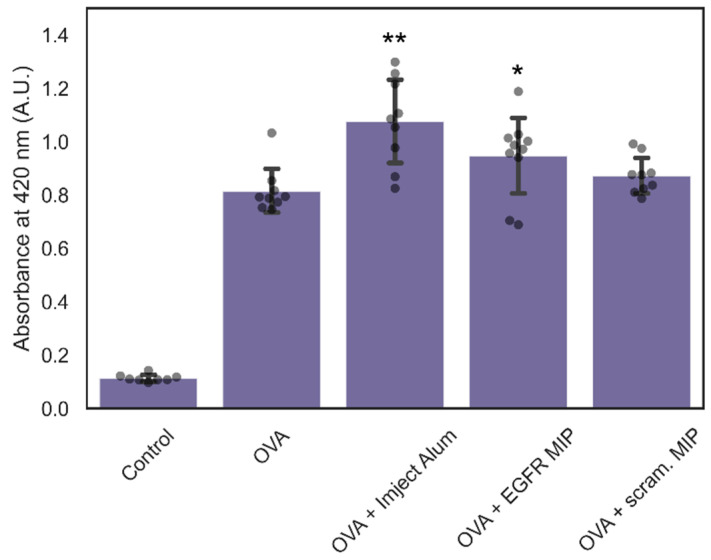
Level of IgG in serum of mice treated with PBS, ovalbumin, a mixture of ovalbumin and Imject Alum Adjuvant, ovalbumin and EGFR-specific nanoMIPs, and ovalbumin and scrambled nanoMIPs. Dots represent IgG levels in individual mouse, each cohort used between 8-10 mice. * *p* < 0.05, ** *p* < 0.01 relative to ovalbumin-administered cohort.

As expected, the highest IgG levels were obtained in mice treated with a mixture of ovalbumin and Imject Alum Adjuvant (*p* < 0.01 relative to ovalbumin alone) (Figure 9). Mice treated with a mixture of ovalbumin and nanoMIPs prepared against the scrambled peptide showed no significant difference, indicating that these MIPs showed no detectable adjuvant properties under these conditions. There was a small but significant difference (*p* < 0.05) between mice treated with ovalbumin alone and those treated with EGFR nanoMIPs. This may be due to the EGFR-specific MIPs having a greater level of interaction with cells and longer retention time, resulting in a more significant immune response. Given the presence of a measurable immune response following treatment with EGFR nanoMIPs, it is important to consider the risk of adjuvant effects for any attempt at targeted delivery of possible immunogens using nanoMIPs. Should a stronger adjuvant effect be desired (as in the case of vaccine development or immunotherapy), it may be possible to incorporate known adjuvants such as aluminium compounds on the surface of the polymer through adsorption or covalent linking.

## 4. Conclusions

This work investigated the biodistribution, clearance, cytotoxicity and adjuvant properties of nanoMIPs, following both oral and intravenous treatment routes. We found that nanoMIPs administered in low dosages either intravenously or orally are not sequestered rapidly by the reticuloendothelial system and can remain in tissues with no apparent major toxic effects. NanoMIPs were able reach every organ tested, including passing through the BBB, and were cleared from the body via both faeces and urine. NanoMIPs imprinted for the cell surface protein EGFR showed weak adjuvant properties for the antigen ovalbumin, whilst those imprinted for a scrambled control peptide showed no adjuvant properties. Though this highlights the risk of potential increase in toxicity of therapeutic payloads delivered via nanoMIPs, it also demonstrates a potential application of nanoMIPs in immunotherapy, where increased immune response is desirable.

Ultimately, more work is required to build our knowledge of the interactions that occur at the bio-nano interface, as well as the relationship between surface chemistry and biodistribution/clearance. This will lead to a more biologically informed design of polymeric nanomedicines that that can be rationalised for clinical translation. Two logical next steps are the assessment of biodistribution/clearance of nanoMIPs prepared against cell surface proteins (particularly cancer biomarkers), and preparation of nanoMIPs for drug delivery. NanoMIPs have already been designed which are capable of selective targeting different type of cells, in particular tumour or senescent cells [52]. Recently, we have shown that nanoMIPs can bind specifically tumour cells by interacting with membrane protein receptors, such as EGFR [10]. Regarding the use of nanoMIPs for drug delivery, the ideal would be generation of nanoMIPs capable of releasing drug cargo, once internalised by targeted cells, and after being exposed to specific conditions (for example, an acidic tumour environment). Numerous methods have been developed for the attachment of therapeutic agents via degradable linkers that cleave under specific cellular conditions which could be used in the formulation of nanoMIPs that can release their payload with variations in pH, redox environment or the presence of certain enzymes [53]. As with other polymeric drug delivery systems, nanoMIPs require further analysis of their biodistribution, degradation mechanisms and component toxicity before they can be considered for clinical applications. We believe, however, that nanoMIPs capable of targeting different membrane proteins and coupled with bioactive or immunogenic components will soon be powerful tools for targeted drug delivery.

## Data Availability

Data presented in this study are available on request from the corresponding author.

## Supplementary Materials

The following supporting information can be downloaded at: https://www.mdpi.com/article/10.3390/polym14214582/s1, Figure S1. TEM image of trypsin-specific nanoMIPs; Figure S2. Presence of nanoMIPs in tissue samples; (A) intestine control, (B) intestine, OR-100 at 24 h; (C) intestine OR-200 at 2 h. Figure S3. Concentration of nanoMIPs in rat excretion for groups IV-200 and OR-200. (A) Concentration of nanoMIPs in urine; (B) Concentration of nanoMIPs in faeces. Figure S4. (A) CRP concentration in blood of groups IV-200 and OR-200. Dotted lines depict range of basal CRP concentrations in healthy rats; (B) IL-1β concentration in blood of groups IV-200 and OR-200.
