# Supplementary material for: Assessing the In Vivo Biocompatibility of Molecularly Imprinted Polymer Nanoparticles
Source: Polymers (Basel). 2022 Oct 28;14(21):4582. doi: 10.3390/polym14214582 (PMC9655879; doi:10.3390/polym14214582)
Supplement: Supplementary file 1 [file polymers-14-04582-s001.zip › polymers-1986372-supplementary.pdf]

Supplementary Materials

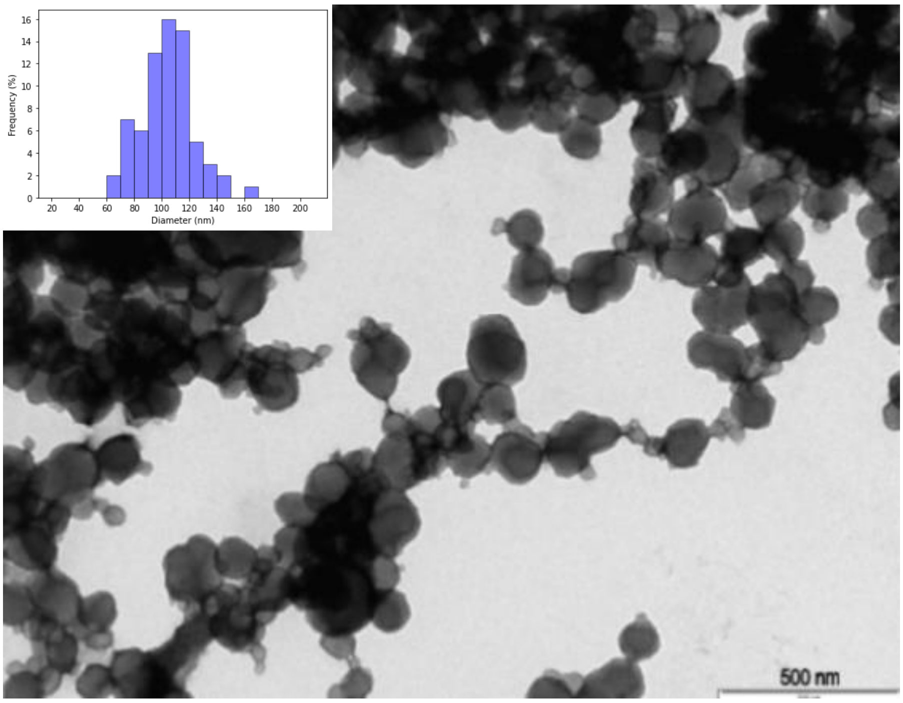

Figure S1. TEM image of trypsin-specific nanoMIPs. Reproduced from Nanoscale Adv., 2019, 1, 3709 with permission from the Royal Society of Chemistry [19].

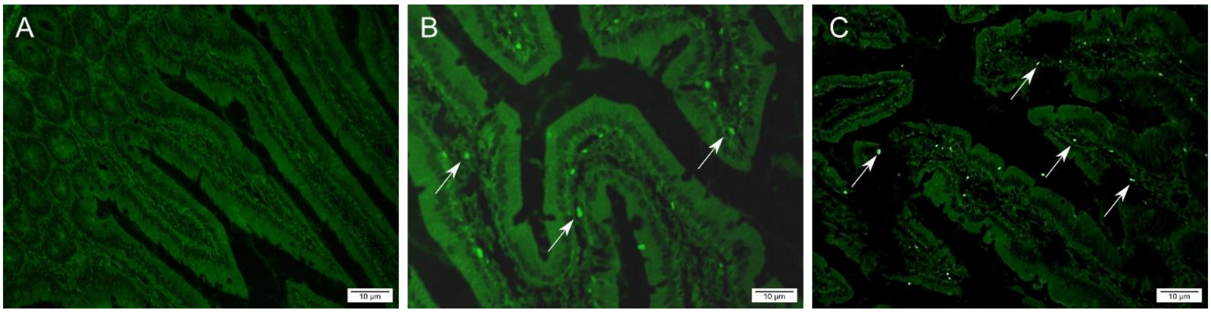

Figure S2. Presence of nanoMIPs in tissue samples; (**A**) intestine control, (**B**) intestine, OR-100 at 24 h; (**C**) intestine OR-200 at 2h. Scale bar = 10 µm.

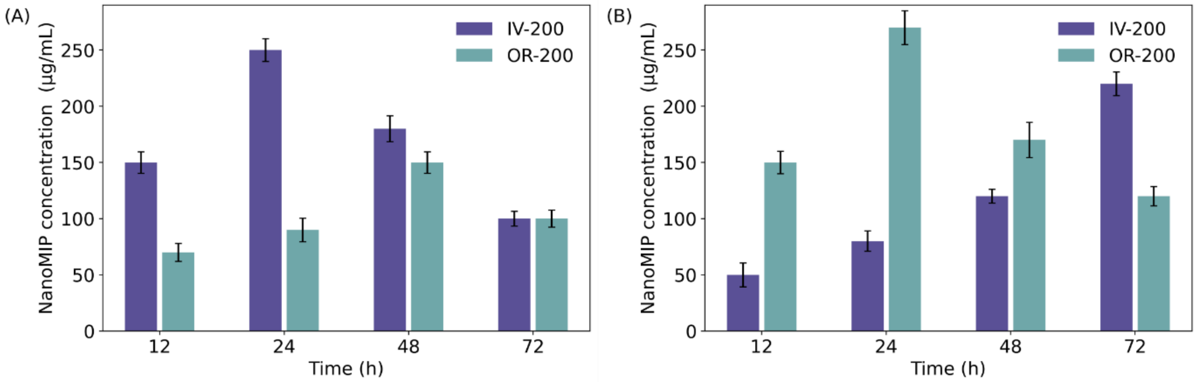

Figure S3. Concentration of nanoMIPs in rat excretion for groups IV-200 and OR-200. (**A**) Concentration of nanoMIPs in urine; (**B**) Concentration of nanoMIPs in faeces.

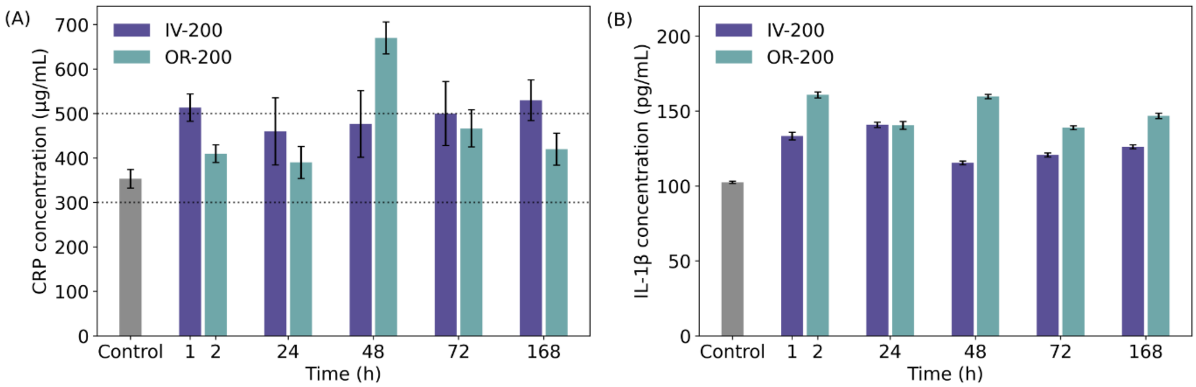

Figure S4. (**A**) CRP concentration in blood of groups IV-200 and OR-200. Dotted lines depict range of basal CRP concentrations in healthy rats; (**B**) IL-1β concentration in blood of groups IV-200 and OR-200.
